# Decreased Plasma Nesfatin-1 Level Is Related to the Thyroid Dysfunction in Patients with Type 2 Diabetes Mellitus

**DOI:** 10.1155/2014/128014

**Published:** 2014-06-04

**Authors:** Fupeng Liu, Qing Yang, Ning Gao, Fangfang Liu, Shaohua Chen

**Affiliations:** ^1^Department of Endocrinology, Central People's Hospital of Tengzhou City, Tengzhou, Shandong 277500, China; ^2^Department of Medicine, Maternal and Child Care Service Centre of Tengzhou City, Tengzhou, Shandong 277500, China; ^3^Department of Endocrinology, Qianfoshan Hospital of Shandong University, Jinan, Shandong 250000, China

## Abstract

*Aims*. Thyroid dysfunction is frequently observed in patients with type 2 diabetes mellitus (T2DM), but the underlying mechanism is still poorly understood. The present study aimed to investigate whether nesfatin-1 played a role in the thyroid dysfunction in patients with T2DM. *Methods*. 55 euthyroid patients were enrolled in this study including 30 patients with T2DM and 25 patients with impaired glucose regulation (IGR). 30 age-matched healthy people were also included as the control. The plasma levels of nesfatin-1, thyrotropin (TSH), and glycosylated hemoglobin (HbA1c) as well as the body mass index (BMI) were comparatively analyzed among the three groups. *Results*. The nesfatin-1 was significantly lower in patients with T2DM than in patients with IGR and in the control. On the contrary, the TSH level was significantly higher in patients with T2DM than in patients with IGR and in the control. Simple regression analysis showed that the plasma nesfatin-1 was negatively correlated with the TSH and HbA1c levels and positively correlated with the BMI. With multiple stepwise regression analysis, the nesfatin-1 remained to be independently correlated with the TSH, BMI, and HbA1c. *Conclusions*. The study was suggesting a role of nesfatin-1 in thyroid dysfunction in patients with T2DM.

## 1. Introduction

Thyroid dysfunction is frequently observed in patients with type 2 diabetes mellitus (T2DM), but the underlying mechanism is still poorly understood [1–3]. Nesfatin-1 is a peptide that has been identified in the hypothalamus including the paraventricular nucleus (PVN) and is encoded in the N-terminal region of the precursor protein NEFA/nucleobindin-2 (NUCB2) [4]. A major function of the nesfatin-1 is inhibition of food intake and regulation of blood glucose in time-, dose-, and insulin-dependent manners [4–7]. Studies have shown that nesfatin-1 is colocalized with thyrotropin-releasing hormone (TRH) and affects the membrane potential of TRH neurons in the PVN, which is known to be closely related to the regulation of thyroid function [8–10]. Also interesting, Sawicka et al. have reported that the unusual thyroid hormone level is associated with the change of ghrelin expression, while ghrelin has been shown to be coexpressed with nesfatin-1 in gastric X/A-like endocrine cells [11, 12]. These findings indicate that nesfatin-1 may have a close relationship with thyroid function. In the present study, we explored the relationship between nesfatin-1 and thyroid dysfunction in patients with T2DM by comparing the plasma levels of nesfatin-1 and TSH between the patients with T2DM and those with impaired glucose regulation (IGR) but without thyroid diseases. Healthy people with normal glucose tolerance were used as the control. Our results suggested that the abnormal plasma level of nesfatin-1 was associated with the dysfunction of thyroid in patients with T2DM.

## 2. Materials and Methods

### 2.1. Patients

A total of 55 patients with abnormal glucose metabolism were enrolled in this study, including 30 patients with T2DM and 25 patients with IGR. All patients were randomly selected from those who visited the Outpatient Department of Qianfoshan Hospital, Shandong University, between February and September 2012. T2DM and IGR were diagnosed with the 75 g oral glucose tolerance test according to the definition given by World Health Organization in 1999. The IGR patients were all newly diagnosed and did not receive any diabetes medications or diets before. The mean age and body mass index (BMI) of the T2DM patients were 62.23 ± 8.11 years old and 25.79 ± 2.64 kg/m^2^, respectively. The mean age and BMI of the IGR patients were 60.04 ± 9.60 years old and 25.76 ± 2.86 kg/m^2^, respectively. There were no significant differences for the age and BMI between T2DM and IGR patients. 30 age-matched healthy people (age, 59.13 ± 9.45 years; BMI, 23.71 ± 2.65 kg/m^2^) who visited our hospital for regular physical examination were selected as the controls. All the control people had a normal glucose tolerance, had no family history of diabetes or other endocrine disorders, and were not taking any medications known to change glucose tolerance. Clinical exclusion criteria included type 1 diabetes mellitus (T1DM), history of thyroid and other endocrine disorders, taking any drugs known to alter the thyroid function (e.g., antihyperthyroidism drugs, thyroxine, etc.) in the past two months, acute diabetes complications, severe infectious disease, pregnancy, surgery or trauma in recent times, heart failure, hypertension, liver or kidney disease, and cancer. All people in this study were Han Chinese. The present study was conducted based on the principles of the Declaration of Helsinki and approved by the Ethics Committee of Shandong University. Informed consent was obtained from all people in this study.

### 2.2. Clinical and Laboratory Examination

Physical examination of the patients including their height, weight, and blood pressure was performed in the morning before breakfast. The BMI was calculated with the formula of “BMI = weight (kg)/(height (m))^2^.” Fasting blood samples (12 h overnight fast) were collected at the same time for measurements of fasting blood glucose (FBG), fasting insulin (FINS), glycosylated hemoglobin (HbA1c), triglycerides (TG), total cholesterol (TC), high density lipoprotein cholesterol (HDL-C), low density lipoprotein cholesterol (LDL-C), free triiodothyronine (FT3), free thyroxine (FT4), thyrotropin (TSH), thyroid peroxidase antibody (TPOAb), and thyroglobulin antibody (TGAb). The fasting plasma level of nesfatin-1 was measured with the enzyme-linked immunosorbent (EIA) assay kit according to the manufacturer's instruction (Phoenix Pharmaceuticals, Belmont, CA). The EIA kit had high specificity to nesfatin-1 and no cross-reactivity with ghrelin, angiotensin, visfatin, and NUCB2. The sensitivity of this assay was 0.78 ng/mL and the intra- and interassay coefficients of variation were 10% and 15%, respectively. The homeostasis model assessment of insulin resistance (HOMA-IR) was calculated according to the equation: HOMA-IR = fasting insulin (*μ*U/mL) × fasting glucose (mmol/L)/22.5.

### 2.3. Oral Glucose Tolerance Test

Venous blood samples from each participant were collected at 0, 60, 120, and 180 min, respectively, after glucose load. The results were interpreted according to the definition of World Health Organization Expert Committee on Diabetes Mellitus in 1999: FBG < 6.1 mmol/L and 2 h postprandial blood glucose (2hPG) < 7.8 mmol/L for normal glucose tolerance; FBG > 7.0 mmol/L and 2hPG > 11.1 mmol/L for T2DM; 2hPG < 7.8 mmol/L, FBG > 6.1 but < 7.0 mmol/L for impaired fasting blood glucose; and FBG < 6.1 mmol/L and 2hPG > 7.8 mmol/L but < 11.1 mmol/L for impaired glucose tolerance.

### 2.4. Statistical Analysis

The statistical analyses were performed using the SPSS11.0 software (SPSS Inc., Chicago, IL), and the *P* value of less than 0.05 (two-tailed) was considered to be statistically significant. All data were expressed as the mean ± SD. One-way ANOVA with post hoc (least significant difference) analysis was used to compare mean values among the T2DM patients, IGR patients, and normal controls. Simple and multiple linear regression analyses were used to examine the relationships between the levels of fasting plasma nesfatin-1, thyroid related hormones, and other variables.

## 3. Results

**Figure 1:**
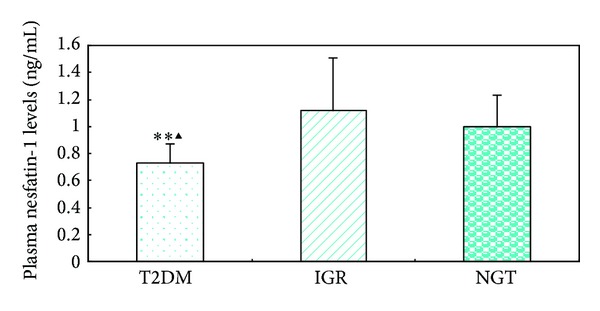
The plasma nesfatin-1 levels in patients with type 2 diabetes mellitus (T2DM) and patients with impaired glucose regulation (IGR) as well as in the control people with normal glucose tolerance (NGT). T2DM versus IGR group, ▲< 0.01; T2DM versus NGT group, ∗∗< 0.01.

Clinical characteristics and laboratory findings of the three groups were shown in Table 1. The fasting plasma level of TSH was significantly higher in the T2DM group than in the IGR and control groups (2.10 ± 1.09 versus 1.50 ± 0.73 versus 1.46 ± 0.60 *μ*IU/mL, both *P* < 0.05). There were no significant differences for other thyroid hormones and antibodies between the T2DM and other two groups. In contrast to TSH, the plasma nesfatin-1 level in the T2DM group was significantly lower than in the groups of IGR and controls (0.73 ± 0.14 versus 1.12 ± 0.39 versus 1.00 ± 0.23 ng/mL, both *P* < 0.01) (Figure 1). No significant difference was obtained for the nesfatin-1 level between the IGR and control groups (Figure 1). Patients with T2DM and patients with IGR had an increased level of BMI, FBG, FINS, and HOMA-IR, compared with the control group (all, *P* < 0.05). The levels of FBG, HOMA-IR, HbA1c, TG, and LDL-C were all significantly higher in the T2DM than in the IGR patients (all, *P* < 0.05). Varieties of FT3, FT4, TPOAb, and TGAb remained consistent among the three groups.

**Figure 2:**
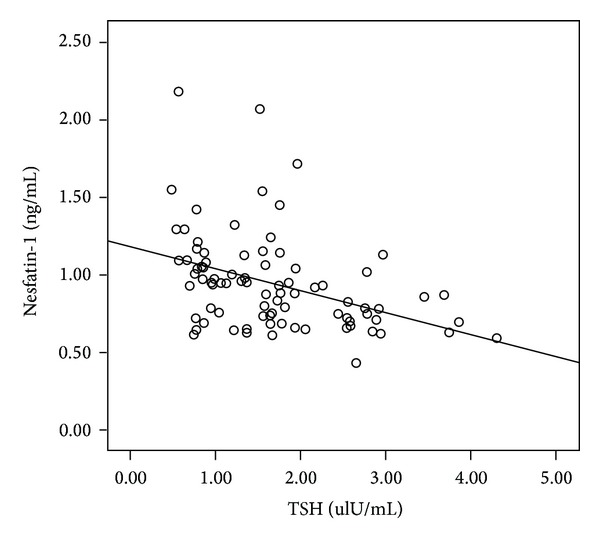
Negative correlation of the plasma nesfatin-1 level with the plasma TSH level (*r* = −0.404, *P* < 0.01).

Simple regression analysis with the pooled data showed that the plasma nesfatin-1 level was positively correlated with the BMI (*r* = 0.397) and negatively correlated with the TSH and HbA1c levels (*r* = −0.404 and −0.389, resp.). The nesfatin-1 level remained independently correlated with the TSH, BMI, and HbA1c levels with multiple stepwise regression analysis (Figure 2). The multiple regression equation was as follows: *Y* = −0.100*X*_TSH_ + 0.049*X*_BMI_ − 0.087*X*_HbA1c_ + 0.405.

## 4. Discussion

In the present study, we found that the plasma TSH level was significantly higher, whereas the plasma nesfatin-1 level was remarkably lower in patients with T2DM, compared with the patients with IGR and the control group. With simple and multiple stepwise regression analyses, the plasma nesfatin-1 level was independently correlated with the TSH level. These results suggested that decreased nesfatin-1 level might play a role in the thyroid dysfunction in T2DM patients.

Thyroid dysfunction is a common endocrine disorder, frequently induced by autoimmune processes, while the patients with diabetes often exhibit thyroid dysfunction. Studies by Radaideh et al. have shown that the overall prevalence of thyroid dysfunction is up to 12.5% in T2DM patients, in contrast to 6.6% in healthy population [13]. The most common disease of thyroid is the subclinical hypothyroidism. Sowiński et al. have demonstrated that about 15% of T2DM patients suffer from overt hypothyroidism, and further 10% present subclinical hypothyroidism [14]. However, the underlying mechanism for the thyroid dysfunction in T2DM patients remains poorly understood, although T1DM and thyroid dysfunction are known to share the similar genotypic milieu [15]. A potential mechanism might be the complex interaction of signaling pathways associated with the glycometabolism and energy metabolism, and the 5′ adenosine monophosphate-activated protein kinase (AMPK) might be a crucial target, which not only participated in the modulation of insulin sensitivity but also is involved in the feedback of thyroid hormones on appetite and energy expenditure [16–18].

Nesfatin-1 is an adipocytokine. In addition to its effect on metabolic regulation and food intake, animal studies have also suggested that nesfatin-1 could enhance insulin release and lead to a time-, dose-, and insulin-dependent reduction of blood glucose [6, 19, 20]. Yang et al. have shown that the central nesfatin-1 can increase the insulin receptor/insulin receptor substrate-1/AMPK/Akt/target of rapamycin complex 2 phosphorylation, resulting in an increase in Fos immunoreactivity in the hypothalamic nuclei [21]. These results suggest that nesfatin-1 may take part in the regulation of both glycometabolism and thyroid hormone functions. Also, the nesfatin-1 immunopositive neurons have been reported to be colocalized with TRH neurons in the PVN and the central nesfatin-1 affects the membrane potential of TRH neurons [8, 22]. Thyroid hormones are tightly regulated by the hypothalamus-pituitary-thyroid axis, and a major component of this control system is the hypothalamic PVN, which contains neurons that produce TRH to regulate the TSH secretion in the anterior pituitary [9]. In the present studies, the plasma nesfatin-1 level was found to be negatively and independently correlated with the TSH level in patients with T2DM. Considering the crucial role of nesfatin-1 in energy and glucose metabolism, we speculated that it might act as a regulatory factor of thyroid function by adjusting AMPK activity and TRH secretion in T2DM patients, thereby resulting in the subclinical hypothyroidism. For the limitation of patients enrolled, we could not analyze whether decreased plasma nesfatin-1 level is related to hyper- or hypothyroidism. It is worthy of a further research.

Our finding of low plasma nesfatin-1 level in T2DM patients was consistent with other studies showing that circulating nesfatin-1 level is dramatically reduced in T2DM patients [23]. We did not obtain a difference for the nesfatin-1 level between IGR and control groups, even though it was slightly increased in the IGR group. This finding was different from the result obtained by Zhang et al. showing that the nesfatin-1 concentration is elevated significantly in patients either with IGT or with newly diagnosed T2DM [24]. Patients' selection may be a possible reason for this discrepancy. Our studies further showed that the plasma nesfatin-1 level was positively correlated with the BMI and negatively correlated with the HbA1c level independently. The present study supports that nesfatin-1 may play a role in the thyroid dysfunction in patients with T2DM.

## Figures and Tables

**Table 1:** Clinical characteristics of the patients ($\overset{-}{x}\pm s$).

Group	T2DM	IGR	NGT
Age (year)	61.23 ± 8.83	59.04 ± 9.01	59.60 ± 9.26
Number (M/F)	30 (16/14)	25 (13/12)	30 (14/16)
Body mass index	26.11 ± 2.55*	26.12 ± 2.07*	24.72 ± 2.48
Duration of diabetes (year)	9.03 ± 3.48	—	—
Fasting blood glucose (mmol/L)	7.37 ± 1.15^∗∗▲^	5.75 ± 0.54**	5.00 ± 0.46
Fasting insulin (mIU/L)	7.72 ± 0.86**	7.83 ± 3.73**	5.56 ± 1.27
HOMA-IR	2.54 ± 0.55^∗∗▲^	1.99 ± 0.92**	1.24 ± 0.33
HbA1c (%)	7.23 ± 1.27^∗∗▲^	5.64 ± 0.42	5.63 ± 0.61
Triglycerides (mmol/L)	2.78 ± 1.71^∗∗△^	1.79 ± 1.24	1.57 ± 1.09
Total cholesterol (mmol/L)	5.56 ± 1.14	5.56 ± 1.09	5.22 ± 0.99
High density lipoprotein cholesterol (mmol/L)	1.43 ± 0.29	1.43 ± 0.26	1.35 ± 0.37
Low density lipoprotein cholesterol (mmol/L)	3.21 ± 0.90^△^	3.78 ± 0.78*	3.08 ± 1.28
Free triiodothyronine (pmol/L)	4.72 ± 0.63	5.04 ± 1.05	5.07 ± 0.74
Free thyroxine (pmol/L)	16.87 ± 2.45	16.85 ± 2.35	17.21 ± 1.58
Thyrotropin (uIU/mL)	2.10 ± 1.09^∗∗△^	1.50 ± 0.73	1.46 ± 0.60
Thyroid peroxidase antibody (pmol/L)	1.93 ± 1.67	1.65 ± 1.71	1.59 ± 1.67
Thyroglobulin antibody (pmol/L)	1.64 ± 0.78	1.50 ± 1.12	1.81 ± 2.44

Data are expressed as mean ± SD. T2DM: type 2 diabetes mellitus; IGR: impaired glucose regulation; NGT: normal glucose tolerance; HOMA-IR: HOMA-insulin resistance index; HbA1c: glycosylated hemoglobin; ∗ < 0.05, ∗∗ < 0.01 versus NGT group; △ < 0.05, ▲ < 0.01 versus IGR group.
